# The BASIDIN effector of the fungus *Moniliophthora perniciosa* promotes positive effects on the seed germination and seedlings development of *Lactuca sativa*

**DOI:** 10.3389/fpls.2025.1529096

**Published:** 2025-01-30

**Authors:** Keilane Silva Farias, Monaliza Macêdo Ferreira, Ivina Barbosa De Oliveira, Ronaldo José Durigan Dalio, Carlos Priminho Pirovani

**Affiliations:** ^1^ Departamento de Ciências Biológicas (DCB), Centro de Biotecnologia e Genética (CBG), Universidade Estadual de Santa Cruz (UESC), Ilhéus, Brazil; ^2^ Centro de Citrucultura Sylvio Moreira, Laboratório de Biotecnologia, Instituto Agronômico, Cordeirópolis, São Paulo. IdeeLab Biotecnologia, Piracicaba, Brazil

**Keywords:** elicitor, germination, lettuce, plant defense, seed, vigor

## Abstract

Plant resistance inducers that activate plant defense mechanisms may be useful in reducing agrotoxic use. Lettuce is among the most economically important leafy vegetable crops in the world. Since lettuce propagates through seeds, the use of high-quality seeds is extremely important for establishing the crop. Several studies have demonstrated the potential of alternative methods of seed treatment with the aim of increasing productivity. Based on this premise, we tested the effect of the rBASIDIN effector regarding its ability to induce germination and physiological changes in lettuce seedlings through seed treatment. The seeds were treated for 30 min by soaking with 50 µg mL^-1^, 75 µg mL^-1^ and 100 µg mL^-1^ of the recombinant effector protein rBASIDIN. Seeds treated with distilled water and 10 mmol of Tris-HCl served as controls. The physiological parameters evaluated were germination percentage at 4 and 7 days, seedling length (aerial part and root), dry and fresh mass, electrical conductivity, and enzymatic activity. Seeds treated with 50 and 75 µg mL^-1^ of rBASIDIN germinated earlier than the controls. Treatment with rBASIDIN at a concentration of 50 µg mL^-1^ resulted in seedlings with an average root length of 1.51 cm, while the average lengths of the controls (H_2_O and buffer) were 0.86 and 0.70 cm respectively. Seed treatment with rBASIDIN caused an increase in the fresh and dry weight of the plants. The lowest electrolyte leakage was detected in seeds treated with the three concentrations of rBASIDIN compared to the controls. Regarding the activity of defense enzymes, seedlings treated with rBASIDIN at lower concentrations showed higher chitinase and β-glucanase activity compared to the controls. The results indicated that the rBASIDIN effector plays an important signaling role in lettuce seeds, since small doses are already sufficient to induce changes in physiological parameters to obtain more vigorous plants.

## 1 Introduction

The demand for healthy food produced in ecologically sustainable systems is growing due to greater nutritional, economic and environmental concerns in today’s world (Triches, 2020). This approach is embodied in the principle of ecologically based agriculture, that is, producing food in adequate quantity and quality without causing depletion and pollution of natural resources (Tamburino et al., 2023).

Lettuce (*Lactuca* spp.) is an annual crop that belongs to the Asteraceae (Compositae) family. It is considered one of the most essential commercial vegetable crops globally, used in salads and sandwiches. In addition, it has medicinal properties, including for the treatment of insomnia, neuropathy and rheumatic pain (Hassan et al., 2021). In Brazil, *Lactuca sativa* L. is the main leafy vegetable crop, with great economic and social importance (Guimarães et al., 2019). However, lettuce’s susceptibility to a wide range of bacterial, viral and fungal pathogens presents significant challenges to its cultivation, requiring preventive measures for protection (Wang et al., 2024).

Lettuce cultivation is carried out by propagation using seeds, whose quality, mainly physiological and sanitary, is fundamental for rapid and uniform establishment of plants in the field for successful production (Albornoz et al., 2019). Among the main factors that affect the physiological quality of seeds is the presence of pathogens. Among the damages caused are reductions in germination and seedling vigor (Albornoz et al., 2019; Costa et al., 2022). In view of this, the use of techniques to improve crop performance in the field is increasingly being studied.

The use of high-quality seeds associated with seed treatment can ensure excellent crop performance, bringing greater economic returns to farmers. Seed treatments can be carried out by chemical, physical and biological methods, with chemical treatment being the most commonly used (Javed et al., 2022). However, the indiscriminate use of these chemical products have caused harmful effects on the environment and generated resistance of pathogens to various molecules (Costa et al., 2022).

Thus, alternative treatment methods, such as biological methods, have shown significant results in eliminating or reducing phytopathogens associated with seeds, providing benefits for germination, growth and development of plants, thus boosting productivity, in addition to mitigating negative environmental impacts by reducing the use of agrochemicals (De Britto et al., 2021; Yang et al., 2022; Tamburino et al., 2023).

Seed *priming* is an environmentally friendly biological treatment method, which can effectively induce plant immunological memory, with noteworthy potential for sustainable crop protection (Yang et al., 2022). This method affects various physiological systems in general, both in seeds and plants by promoting faster response of plants to stress through more efficient absorption of nutrients, improving their growth and development (Singh et al., 2021).

Typically, some immune inductive biomolecules derived from pathogens (bacteria, fungi and oomycetes) can be used to activate the plant defense system, increasing resistance to various phytopathogens (Zhu et al., 2024). For example, the bacterial protein harpin can be applied to a variety of non-host plant species, acting as an inducer by activating defense responses against many pathogens, besides promoting plant growth (Sheikh et al., 2019; Zhu et al., 2024). The elicitor protein PevD1 secreted by the fungus *Verticillium dahliae*, when expressed in *Escherichia coli*, can be purified and used in the treatment of tobacco plants, where it induces a hypersensitive response in leaves and promotes production of multiple signaling molecules and secondary metabolites. For example, treatment of plants with recombinant PevD1 promoted increased systemic resistance to tobacco mosaic virus (TMV) compared to control plants (Wang et al., 2012).

Effector molecules can function as elicitors through their interaction with various physiological responses of plants. The effector protein CfPDIP1, secreted by the hemibiotrophic fungus *Colletotrichum falcatum*, can act as an elicitor by triggering defense responses in sugarcane. The foliar priming caused by the recombinant protein suppressed the extent of lesions caused by the pathogen *C. falcatum*, in addition to inducing the systemic expression of defense-related genes with the concomitant reduction of the pathogen biomass (Ashwin et al., 2018).

BASIDIN, identified as a potential effector protein of the fungus *Moniliophthora perniciosa* in its interaction with the cocoa tree *Theobroma cacao* can act as an effector and as an elicitor of defense responses in plants. When the recombinant protein rBASIDIN was sprayed on *Solanum lycopersicum* plants, it interfered with the plants’ defense system, causing wilting, cell death, production of hydrogen peroxide, damage to the leaf membrane and a decrease in the photosynthetic rate (Farias et al., 2023).

Thus, we tested the hypothesis that the BASIDIN effector can be used to activate defense mechanisms in host and non-host plants by stimulating seed germination, seedling development and root growth. In this work, lettuce was chosen as the object of study, since it is among the most consumed leafy vegetables in the world, being a source of nutrients and bioactive compounds valuable for human health. Seed preparation with BASIDIN can be an efficient and economical technique. However, the literature is still very scarce on the beneficial effect of effectors on germination, uniformity and growth of cultivated plants, such as lettuce. Hence, the objective of this work was to evaluate the effect of bio-conditioning lettuce seeds with the protein effector rBASIDIN (recombinant effector protein), on the physiological quality of the seeds and seedlings.

## 2 Materials and methods

### 2.1 Treatment of lettuce seeds with the rBASIDIN effector

A total of 100 seeds of *Lactuca sativa* cv Mimosa lettuce (Category S2, 2018 harvest) were used per treatment, with germination percentage of 89%. They were purchased from an agricultural trading house. The seeds were treated manually by soaking them in various concentrations of the effector protein rBASIDIN (50 µg mL^-1^, 75 µg mL^-1^ and 100 µg mL^-1^) for 30 min. After soaking, the seeds were aseptically air-dried and used throughout the study. Seeds treated with distilled water and Tris-HCl 10 mmol L^-1^ were used as controls. Once this seed treatment process was completed, the physiological and biochemical qualities were evaluated using the following laboratory tests:

#### 2.1.1 Germination test

The germination test was conducted with 100 lettuce seeds for each treatment, divided into four replicates of 25 seeds each, distributed in Petri dishes (100 x 20 mm) on two sheets of germitest paper moistened with a quantity of distilled water equivalent to 2.5 times the mass of the dry paper. The seeds were then incubated in a germination chamber at 25°C with a light/dark photoperiod of 8/16 hours for seven days. A volume of approximately 3 mL of distilled water was applied to all Petri dishes when their moisture content decreased. Seeds were considered germinated when the radicle length exceeded 2 mm, and germination counts were conducted four and seven days after sowing (DAS), according to the criteria established by the Standards for Seed Analysis (Brasil, 2009). The results were expressed as germination percentage. Plant morphological parameters, such as root and shoot length and dry and fresh mass, were determined on the 7th DAS.

#### 2.1.2 Length of the aerial part and primary root of the lettuce seedlings

To measure the average length, 25 normal seedlings from each treatment obtained from the 7-day germination test were measured separately to determine the length of the roots and aerial part, with a ruler. The average seedling length was obtained by adding the measurements of each replicate and dividing by the total number of seedlings, and the results were expressed in cm.

#### 2.1.3 Dry mass and fresh mass of lettuce seedlings

After germination assessment, 50 normal seedlings from each treatment were separated from the cotyledons and weighed on a precision scale to determine fresh matter. After weighing, they were placed in paper bags and placed in a forced-air oven at 60 °C for 72 h. Then, the seedlings were weighed again and the results were expressed as g/seedling.

### 2.2 Determination of electrical conductivity

To determine electrical conductivity, the method proposed by (Veronesi et al., 2014) was employed, with some modifications, using three replicates of 25 seeds per treatment. The seeds were previously weighed with a precision analytical balance (0.0001) and were subjected to imbibition with rBASIDIN at different concentrations (50 µg mL^-1^, 75 µg mL^-1^ and 100 µg mL^-1^) for 30 min. As a control, the seeds were soaked in distilled water and 10 mmol L^-1^ of Tris HCl buffer. Afterward, the seeds were dried with paper towels, transferred to a container containing 10 mL of autoclaved distilled water and kept in a BOD at a temperature of 25 °C. Electrical conductivity was determined after 24 hours using a conductivity meter (CD, Lutron Electronics model 4322), and the results were expressed as µS cm^-1^ g^-1^ of seeds. The conductivity of autoclaved distilled water was measured as a blank control (CO). The calculation of electrical conductivity was determined according to the following equation:


$$EC=\frac{\text{R}ce-\text{CO}}{\text{ms}}$$

Where:

EC= total electrical conductivity, in µS cm^-1^ g^-1^;

Rce = electrical conductivity reading of soaked seeds;

CO= electrical conductivity of distilled water (blank control); and

Ms= mass of 25 seeds, in g^-1^.

### 2.3 Enzymatic activities of lettuce seedlings from seeds treated with rBASIDIN

The activity of enzymes that are part of the plant defense system against the action of pests was evaluated to verify the effect of rBASIDIN as an inducer. To analyze the enzymatic activity of the enzymes chitinase, glucanase and catalase, the lettuce seeds were treated by immersion for 30 min, in each treatment, at the concentrations previously described. Sixty seedlings of each treatment were used (four replicates of 15 seedlings).

Crude enzymatic extracts were obtained by macerating the seedlings in liquid nitrogen, followed by the addition of 1 mL of 0.1 M potassium phosphate buffer, pH 6.8, and ethylenediaminetetraacetic acid (EDTA). The samples were processed in an ultrasonicator (Gex ultrasonic processor model 130, 130W) on ice until the tissue was completely disrupted, with a pulse of 8 s, at intervals of 10 s and an amplitude of 70%. Then the samples were centrifuged for 5 min at 17,530 g. Immediately afterwards, 200 µL aliquots of the aqueous phase were collected and diluted in 800 µL of the appropriate buffer for analysis of the activity of each enzyme. An aliquot of 30 µL of each sample was separated for quantification by the Bradford methods (Bradford, 1976) using BSA as standard. The specifics of the methods for each enzyme are described below.

#### 2.3.1 Chitinase enzyme activity

For the analysis of chitinase activity, the Chitinase Fluorimetric Assay Kit (Sigma-Aldrich) was used according to the manufacturer’s instructions. The test mixture contained 5 µL of seedling extract and 45 µL of substrate solution, which was incubated at 37°C for 30 min. Then the reaction was stopped by adding 100 µL of stop solution (Na_2_CO_3_ at 0.4 mol L^-1^). The substrate solution contained 0.2 mg mL^-1^ of 4-methylumbelliferyl beta-D-N,N’,N’’-triacetylchitotriose or 1 mg mL^-1^ of methylumbelliferyl beta-D-N,N’-diacetylchitobioside hydrate. The assay was based on the enzymatic hydrolysis of chitinase substrates that release 4-methylumbelliferone (4MU). The fluorescence released in the basic medium was measured at an excitation wavelength of 360 ɳm and an emission wavelength of 450 ɳm. The analysis was performed in duplicate, using two substrates in each assay: the substrate 4-Methylumbelliferyl beta-D-N,N’,N’’-triacetylchitotriose for detection of endochitinase activity and methylumbelliferyl beta-D-N,N’-diacetylchitobioside hydrate for detection of exochitinase activity. In each assay, *Trichoderma viride* chitinase was used as a positive control and the blank (substrate only) as a negative control.

#### 2.3.2 β-1-3 glucanase activity

The enzymatic activity of glucanase, a plant defense enzyme that can attack glucans in the pathogen cell wall, was determined by measuring the hydrolysis of laminarin as a substrate, as described by Miller (1959). A reaction solution containing laminarin as substrate (2 mg mL^-1^) and samples of the total extract of lettuce seedlings obtained as described in 2.3, in a ratio of 1:1, was incubated at 40 °C under shaking for 3 h. Then the samples were centrifuged at 1,792 *g* for 5 min and the supernatant was collected. Next, 200 μL of the reaction solution and 100 μL of the 3,5-dinitrosalicylic acid (DNS) solution were placed in new microtubes, which were shaken vigorously, vortexed and placed in a water bath at 100 °C for 5 min. The reaction was then stopped by cooling on ice for about 2 min. The final volume was adjusted to 1000 μL by adding water, and finally, the samples were pipetted in quadruplicate into microplates. Absorbance readings were performed using a wavelength of 550 ɳm with a SpectraMax Paradigm spectrophotometer (Molecular Devices). Controls of each sample containing laminarin, seedling extract and DNS were also read. The glucose standard curve was plotted as a reference. One unit (U) of enzyme activity was defined as the amount of enzyme required to release 1 µmol of glucose per minute.

#### 2.3.3 Catalase activity

CAT activity was determined according to the method described by (Havir and McHale, 1987), with some modifications. The activity was defined by the speed of H_2_O_2_ consumption, in a reaction monitored in the Spectramax Paradigm microplate spectrophotometer (Molecular Devices). The reaction buffer consisted of 50 mmol L^-1^ sodium and 12.5 mmol L^-1^ H_2_O_2_ with 20 µL of crude extract from lettuce seedlings, with the activity carried out at 30 °C. The reaction was initiated by adding H_2_O_2_ at 30 mmol L^-1^, and readings were performed by calculating the decay at 240 ɳm for 300 s against a blank free of crude seedling extract, expressed in µmol H_2_O_2_ min^-1^ mg^-1^ of protein, using a molar extinction coefficient of 36 M^-1^ cm^-1^.

## 3 Results

### 3.1 Effect of rBASIDIN on seed germination

**Figure 1:**
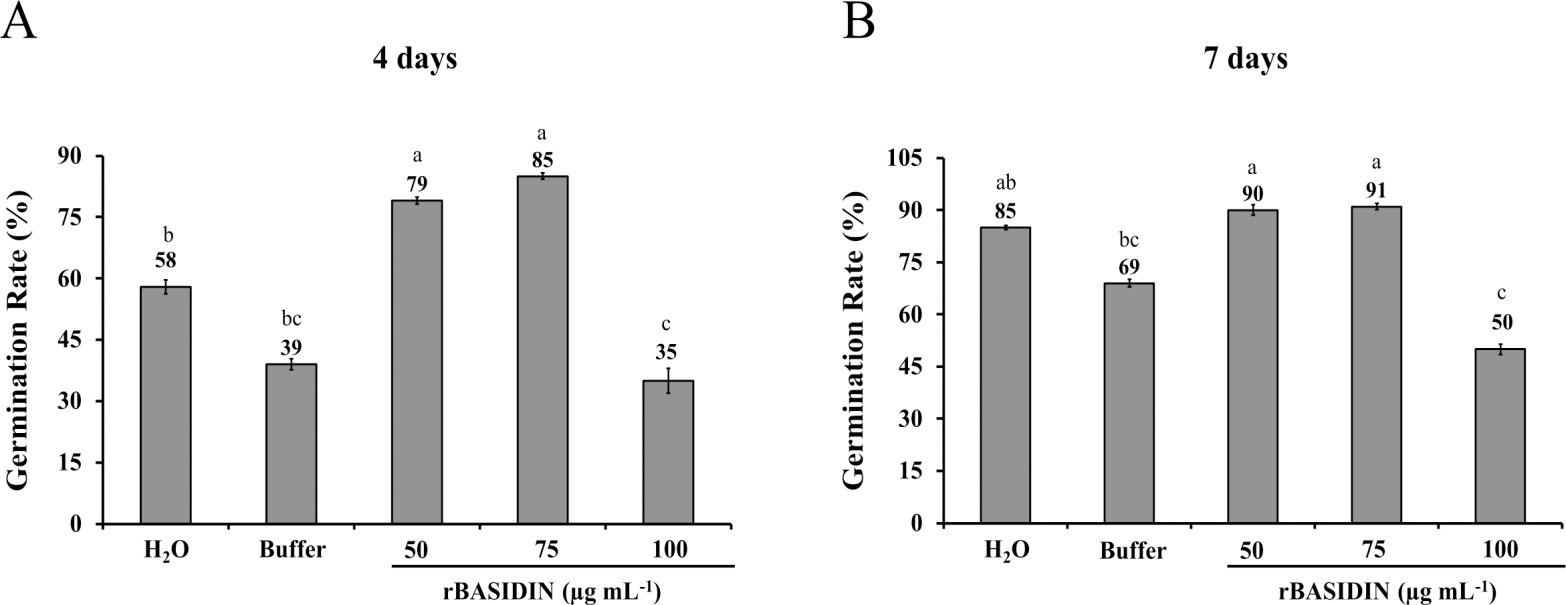
Average germination test data (%) of lettuce seeds treated with different concentrations of rBASIDIN. **(A)** first germination count four days after sowing. **(B)** second germination count seven days of sowing. Averages followed by the same letter do not differ from each other according to the Tukey test at 5% probability.

In the germination test, there was a significant difference (p ≤ 0.05) between treatments, as can be seen in **Figures 1A, B**. After four days of germination, the lettuce seeds that received treatments with rBASIDIN at concentrations of 50 µg mL^-1^ and 75 µg mL^-1^ showed higher germination percentages than those of the controls, reaching average values of 79% and 85% of germinated seeds respectively, while the seeds treated with distilled water showed 58% germination and 39% for buffer exposure (**Figure 1A**). Subsequently, the seeds treated with the rBASIDIN protein at a concentration of 100 µg mL^-1^, had a lower germination percentage, of 35%, compared to the controls (**Figure 1A**). Furthermore, the germination percentage of the lettuce seeds treated with rBASIDIN at concentrations of 50 µg mL^-1^ and 75 µg mL^-1^ at seven days did not differ significantly compared to the control seeds treated with distilled water (**Figure 1B**), but differed from the buffer control treatment and the treatment with rBASIDIN at a concentration of 100 µg mL^-1^ (**Figure 1B**).

**Figure 2:**
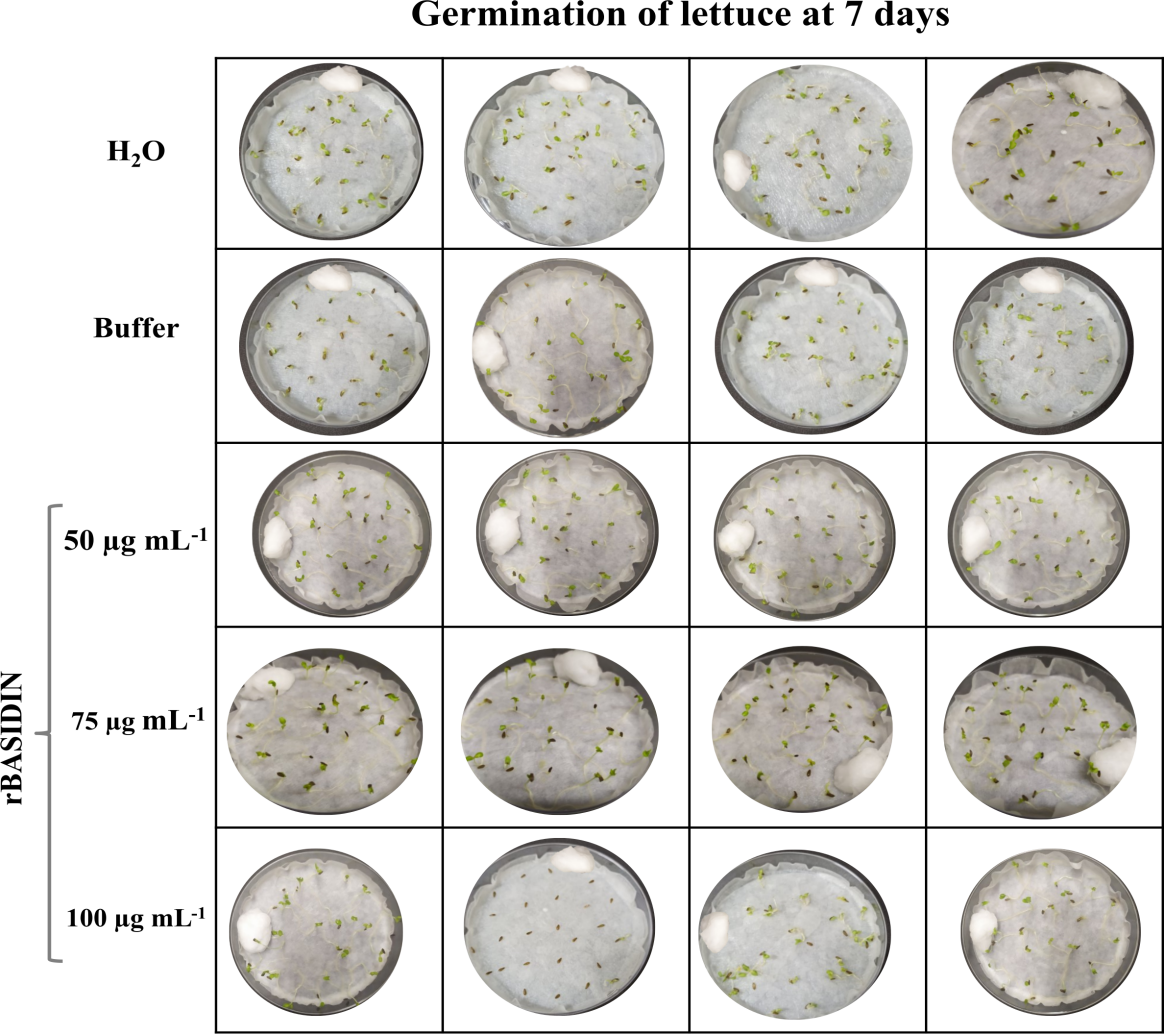
Quality of lettuce seedlings 7 days after the seeds were subjected to treatment with different concentrations of rBASIDIN.

Treatment of lettuce seeds with rBASIDIN at concentrations of 50 µg mL^-1^ and 75 µg mL^-1^ did not hamper seedling development (uniformity and greater formation of normal seedlings) (**Figure 2**).

### 3.2 Effect of rBASIDIN on lettuce seedling length (shoot and root)

**Figure 3:**
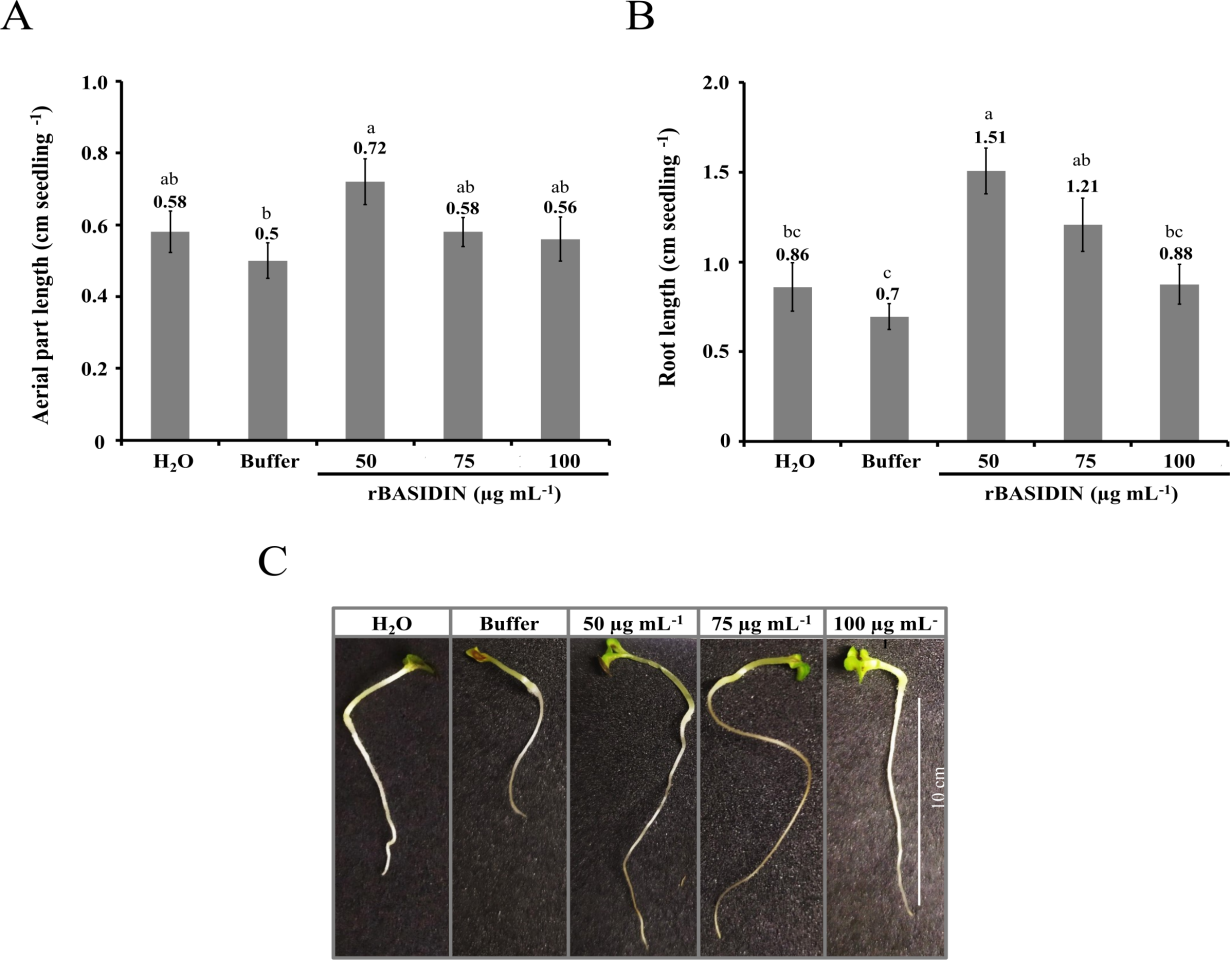
Evaluation of root and shoot length of lettuce seedlings obtained from seeds treated with different concentrations of the effector protein rBASIDIN. **(A)** length of aerial part; **(B)** root length and **(C)** image of seedlings of each treatment. Means followed by the same letter do not differ from each other by the Tukey test (p ≤ 0.05).

There was a greater increase in the length of seedlings obtained from seeds that were treated with rBASIDIN at a concentration of 50 µg mL^-1^ (0.72 cm) in comparison with the other treatments, which presented an average length of 0.58 cm in the treatment with 75 µg mL^-1^, distilled water (0.58 cm), 100 µg mL^-1^ (0.56 cm) and buffer (0.50 cm) (**Figure 3A**). Regarding the length of the primary root, statistical differences were observed between treatments. rBASIDIN at a concentration of 50 µg mL^-1^ presented an average root length of 1.51 cm, which was statically equal to the average length of seedlings obtained from the treatment of seeds with rBASIDIN at a concentration of 75 µg mL^-1^, but statistically different from the other treatments (**Figure 3B**). The largest seedlings were obtained in the treatments with rBASIDIN (**Figure 3C**).

### 3.3 Dry and fresh mass

**Figure 4:**
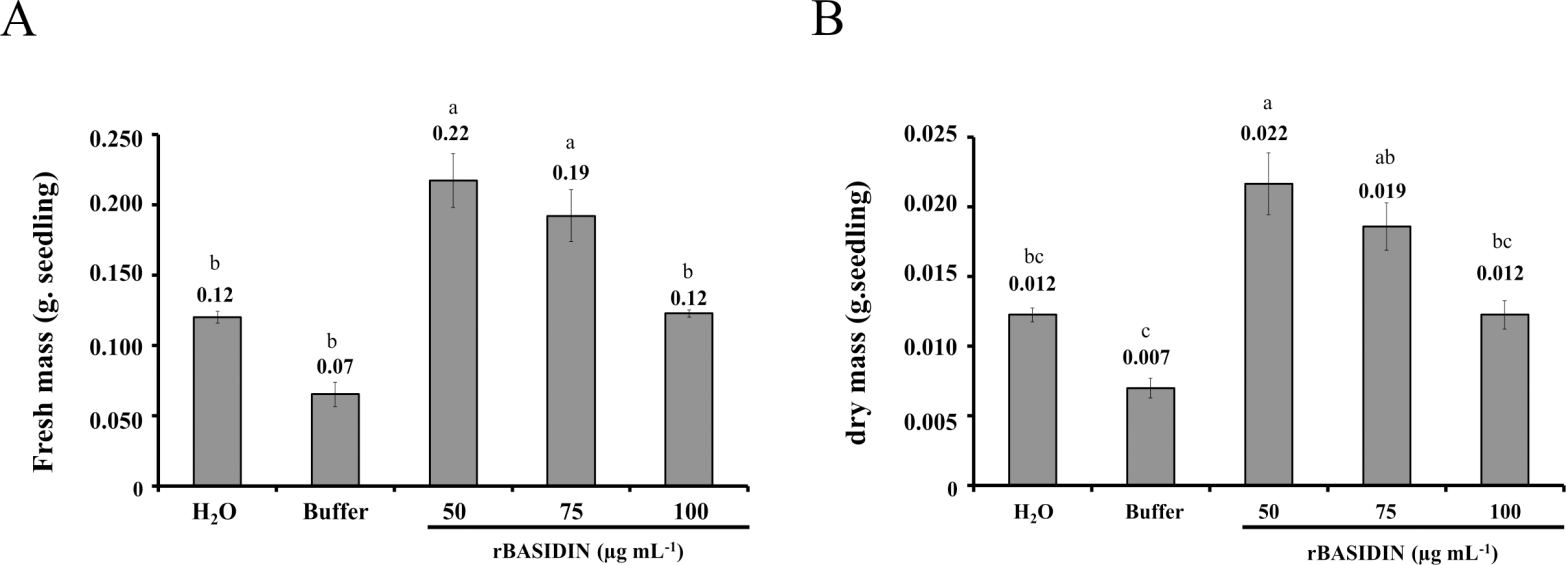
Fresh mass **(A)** and dry mass **(B)** of lettuce seedlings from seeds that were subjected to treatment with different concentrations of rBASIDIN. Values are means of three replicates (n=3). Means followed by the same letter do not differ from each other by the Tukey test (p ≤ 0.05).

The fresh mass of lettuce seedlings varied according to the treatment to which the seeds were submitted. The treatments with rBASIDIN at concentrations of 50 µg mL^-1^ and 75 µg mL^-1^ showed statistically greater fresh mass, with averages of 0.22 g seedling^-1^ and 0.19 g seedling^-1^, respectively (**Figure 4A**). In the other treatments, there was no significant difference, with averages of 0.07 g seedling^-1^ to 0.12 g seedling^-1^.

For dry mass (**Figure 4B**), the plants from seeds treated with rBASIDIN at a concentration of 50 µg mL^-1^ (0.022 g seedling^-1^) were statistically equal to those submitted to 75 µg mL^-1^ (0.019 g seedling^-1^), but differed from the others. Lettuce seedlings from seeds treated with rBASIDIN at a concentration of 75 µg mL^-1^ did not differ from treatments with rBASIDIN at a concentration of 100 µg mL^-1^ (0.012 g seedling^-1^), or the controls, distilled water (0.012 g seedling^-1^) and buffer (0.007 g seedling^-1^).

### 3.4 Effect of exogenous application of rBASIDIN on the electrical conductivity of lettuce seeds

**Figure 5:**
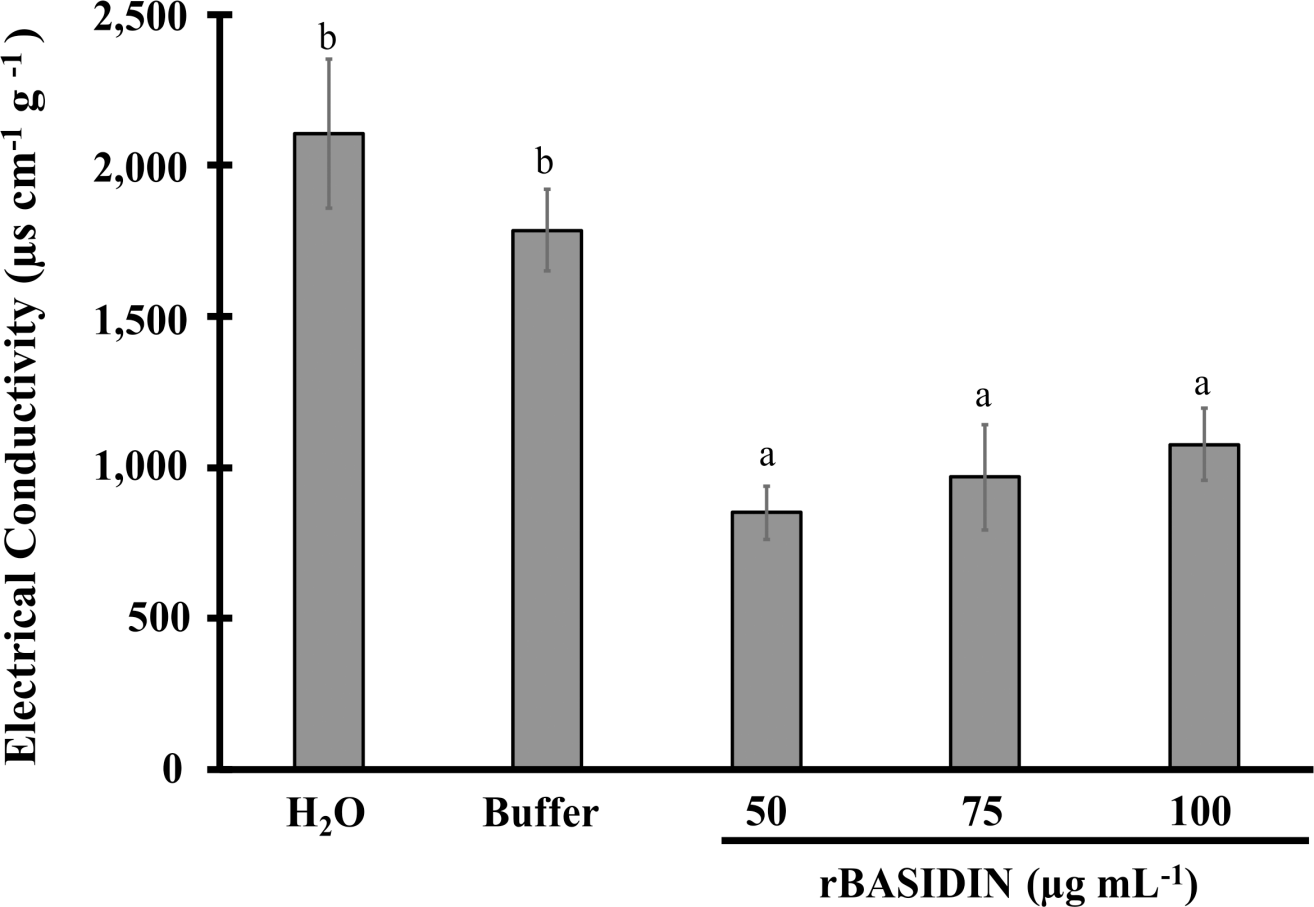
Electrical conductivity of lettuce seeds under the influence of conditioning with different concentrations of rBASIDIN. Means followed by the same letter belong to the same group according to the Scott-Knott cluster test at 5% probability, and do not differ from each other. The bars correspond to the standard error of the mean (n=3).

The electrical conductivity (EC) of the seeds in all treatments with rBASIDIN had lower values compared to the control treatments. Although a linear increase in the amount of electrolytes released by the seeds was observed with increasing rBASIDIN concentration, there was no statistical difference (**Figure 5**). At a concentration of 50 µg mL^-1^, a lower CE occurred, of 851 µS cm^-1^ g^-1^, in relation to the control treatments, water (2,105 µS cm^-1^ g^-1^) and buffer (1,785 µg cm^-1^ g^-1^) (**Figure 5**).

### 3.5 Effect of rBASIDIN on enzymatic activities of lettuce during the seedling stage

**Figure 6:**
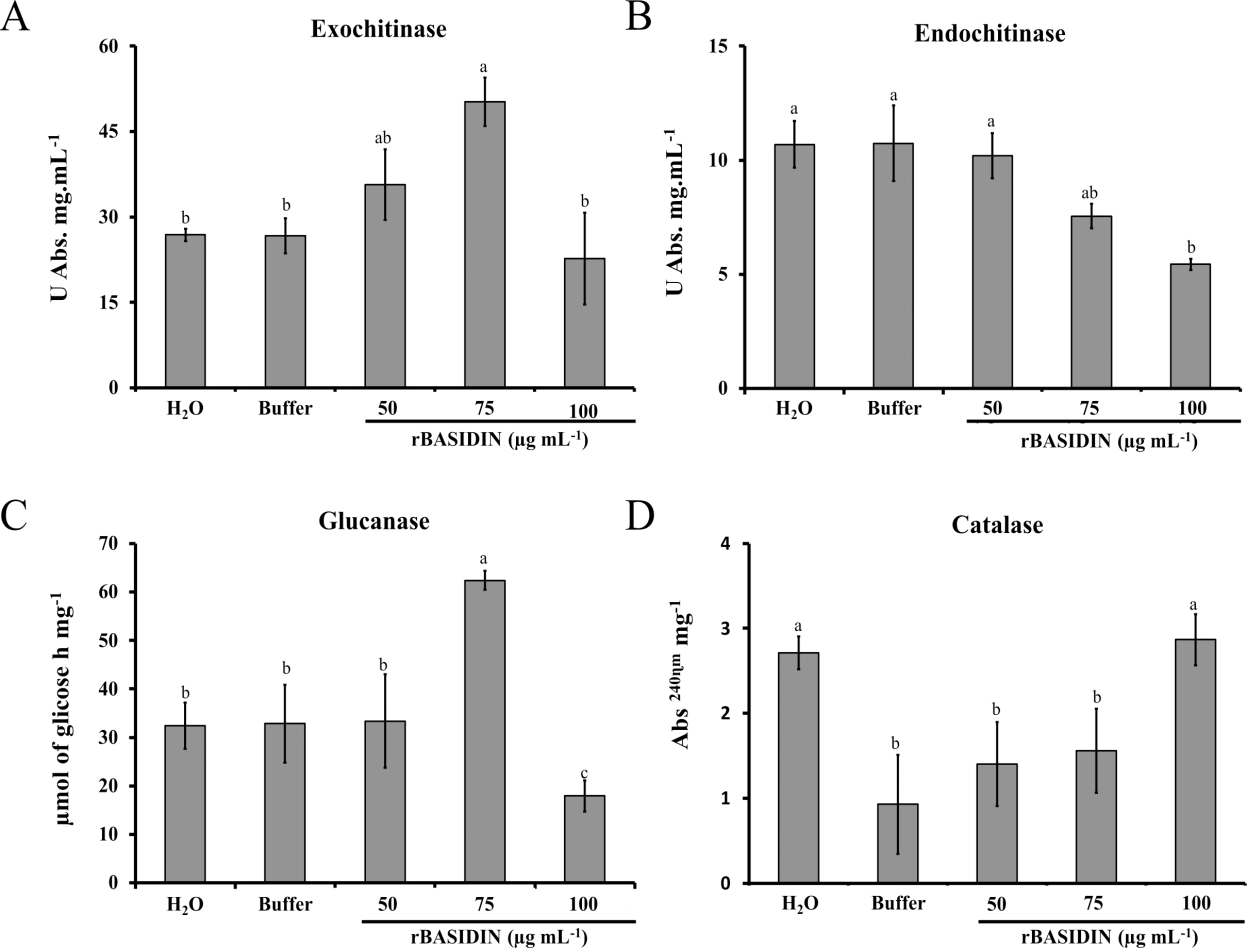
Enzymatic activity in lettuce seedlings from seeds treated with different concentrations of rBASIDIN. **(A)** Exochitinase enzyme activity. **(B)** Endochitinase enzyme activity. Specific activity is expressed in units per milligram of protein (Unit mg protein^-1^). **(C)** β-1,3-glucanase enzyme activity. Activity is expressed in µmol of glucose per hour, per milligram of protein. **(D)**: Catalase enzyme activity is expressed by the absorbance value per milligram of protein. Mean values of four technical replicates (n=4). Means followed by the same letter do not differ from each other according to the Tukey test (p ≤ 0.05). The bars correspond to the standard errors of the means.

The treatment of lettuce seeds with different concentrations of rBASIDIN stimulated the enzymatic activity positively or negatively depending on the concentration used. When lettuce seeds were treated with rBASIDIN at a concentration of 75 µg mL^-1^, the seedlings showed an increase in exochitinase activities (**Figure 6A**). For seeds that were treated with rBASIDIN at a concentration of 100 µg mL^-1^, there was a sharp decrease in enzyme activity in seedlings, with activity still lower than that of control treatments (**Figure 6A**). For endochitinase activity, rBASIDIN interfered negatively, as can be seen in **Figure 6B**. The concentrations of 75 µg mL^-1^ and 100 µg mL^-1^ induced lower enzyme activity compared to the controls, and the protein at the concentration of 50 µg mL^-1^ showed no difference in activity compared to the controls (**Figure 6B**).

Regarding the activity of β-1-3 glucanase, the maximum increase in activity was observed in lettuce seedlings from the seeds treated with rBASIDIN at a concentration of 75 µg mL^-1^, which presented enzymatic activity of 62.39 µmol glucose h^-1^ mg^-1^ (**Figure 6C**), a result statically different from the other treatments. For the treatment of seeds with rBASIDIN at a concentration of 50 µg mL^-1^, the enzyme activity in seedlings was 33.39 µmol glucose h^-1^ mg^-1^ which was not significantly different from the control treatments in comparison with the other treatments, except for the treatment with rBASIDIN at a concentration of 100 µg mL^-1^. For the seeds treated with distilled water, the seedlings had activity of 32.44 µmol glucose h mg^-1^, and the buffer was 32.82 µmol glucose h^-1^ mg^-1^. Lower activity of glucanase was observed in the treatment of seeds with rBASIDIN at a concentration of 100 µg mL^-1^ (17.93 µmol glucose h^-1^ mg^-1^).

The activity of the enzyme catalase (CAT) in seedlings from seeds treated with different concentrations of rBASIDIN, was higher than in seedlings seeds treated with rBASIDIN at a concentration of 100 µg mL^-1^, with an average value of 2.88 Abs 240 min^-1^ mg^-1^ protein. The seedlings from seeds treated with rBASIDIN at concentrations of 50 µg mL^-1^, 75 µg mL^-1^ and the control with distilled water had, respectively 1.40; 1.56 and 2.71 Abs^240^ min^-1^ mg ^-1^, while the value for the treatment with buffer was, 0.93 Abs^240^ min ^-1^ mg ^-1^ (**Figure 6D**). Static analysis showed a significant difference only between seed treatments with rBASIDIN at a concentration of 100 µg mL^-1^ in relation to the control treatment with the buffer and treatments with rBASIDIN at concentrations of 50 and 75 µg mL^-1^ (**Figure 6D**).

## 4 Discussion

### 4.1 Pretreatment of lettuce seeds with rBASIDIN increased the germination percentage of lettuce seeds

Seed germination is the first critical stage in the life of plants, since seeds are exposed to a wide range of environmental stresses that can affect their germination. To enhance or preserve the germination and productivity of different crops, some seed treatment methods are used, such as *priming* (Nile et al., 2022), pelletizing (Javed et al., 2022) and seed coating (Paravar et al., 2023).

Seed preparation with biotic and abiotic elicitors results in better germination, and can induce plants’ immunological memory, which is maintained stably throughout the development stages and can also be transmitted over generations (Kalaivani et al., 2016; Nile et al., 2022). In this sense, we tested the effect of the rBASIDIN effector regarding its ability to induce germination and physiological changes in lettuce seedlings through seed treatment.

The average germination percentage of lettuce seeds increased due to treatment with rBASIDIN at low concentrations. This increase was observed in treatments with rBASIDIN at 50 and 75 µg mL^-1^ four days after sowing (**Figure 1A**). The beneficial effect on germination observed for these concentrations may have occurred as a result of the action of rBASIDIN on membrane permeability, since cell membrane homeostasis is a prerequisite for normal germination and good physiological functioning of seeds (Dhaliwal and Angeles-Shim, 2022).

The first germination count is an indication of the seeds’ vigor, since seeds that germinate more quickly and produce a higher percentage of normal seedlings are predisposed to be more vigorous (Brasil, 2009). Thus, the treatment of seeds with BASIDIN at the lowest concentrations produced seedlings with uniform development (**Figure 2**). This is an important characteristic since uneven growth of seedlings results in yield losses (Ebone et al., 2020).

Still in this context, at the end of the germination test (seven days after sowing), the seeds treated with the rBASIDIN effector at the concentrations mentioned above presented a germination percentage in accordance with the percentage established by the batch manufacturer (**Figure 1A**). This fact, added to the early germination of seeds on the fourth day, showed that these concentrations were efficient in increasing seed germination. In line with our results, other studies have shown positive results from the use of elicitor/effector molecules derived from pathogens for seed treatment. The use of trehalose, a mycogenic elicitor isolated from the cell wall of *Thrichoderma atroviride* in broccoli seeds provided a remarkable increase of 94% in the seed germination rate (De Britto et al., 2021). The biostimulant PSP1 formulated based on an elicitor protein from the fungus *Sarocladium strictum* demonstrated promising results for the germination potential of wheat seeds in field tests (Martínez et al., 2024).

Increasing the concentration of rBASIDIN used to 100 µg mL^-1^ caused a decrease in the germination rate, both in the first count (four days) and in the second (seven days). This suggests that this dose may have caused a toxic effect on the seed embryo. Other researchers have reported the need for caution when choosing treatment concentrations for seed conditioning (Mahesh et al., 2017; Vázquez-Hernández et al., 2019; De Britto et al., 2021).

Therefore, our data indicate that priming of lettuce seeds with the *M. perniciosa* effector rBASIDIN at appropriate concentrations can induce physiological and biochemical changes that stimulate seeds to germinate faster and increase the germination percentage of *Lactuca sativa* cv (mimosa lettuce).

### 4.2 The effector rBASIDIN has a positive effect on lettuce seedling development

Seedlings emerging from seeds treaed with rBASIDIN at low concentrations showed more promising growth than control seedlings originating from seeds treated with distilled water and buffer. For seedling growth, a significant effect of seed pretreatment with the effector at a concentration of 50 µg mL^-1^ was observed, with average shoot length of 0.72 cm and root length of 1.51 cm (**Figures 3A, B**). It is possible to hypothesize that metabolic events caused by rBASIDIN, at this concentration in the seeds triggered better root and shoot growth, by positively influencing the physiological quality of the seedlings, assuming that the seedlings that grow longer are more vigorous (Kalaivani et al., 2016).

In the present study, an increase in the dry and fresh mass of seedlings that developed from seeds treated with rBASIDIN was also observed, depending on the concentration (50 and 75 µg mL^-1^). This shows that seedlings from these treatments can become more vigorous and productive, since in lettuce crops, fresh matter production is directly related to the plant’s leaf area (Moura Neto et al., 2022). Likewise, a comparable increase in dry and fresh mass parameters was observed in pepper seedlings after seed treatment with abiotic elicitors (Abhayashree et al., 2016).

The use of effectors in other studies has demonstrated positive effects on the parameters analyzed here. For example, the use of the harpin effector HpaXpm in *Arabidopsis thaliana* seeds promoted better root development and higher fresh mass content of the plants I comparison with the control treatment (Liu et al., 2020). However, our results demonstrate great potential of using rBASIDIN to obtain more vigorous and anatomically developed seedlings. However, a field study is necessary to verify whether these qualities remain in an uncontrolled environment.

### 4.3 rBASIDIN induces lower electrical conductivity of lettuce seeds

The electrical conductivity test allows assessing the integrity of cell membranes when seeds are soaked in water. The lower the conductivity value, the better the seed vigor will be, indicating that the cell membranes are intact. On the other hand, a high result indicates that the seeds are deteriorated and have low vigor (Veronesi et al., 2014). Hence, this test can also be used to verify whether the application of pretreatment to seeds before sowing causes any damage, since it allows detecting the deterioration process (Vashisth and Nagarajan, 2010; Adetunji et al., 2021).

Thus, in the electrical conductivity (EC) analysis of lettuce seeds treated with rBASIDIN, we observed that for all concentrations of the effector used, there was a decrease in solute leakage in relation to the control treatments, thus presenting a lower EC at the concentration of 50 µg mL^-1^ (**Figure 6**). This result suggests that rBASIDIN does not damage the membrane integrity of lettuce seeds. The reduction of electrolytes in seeds treated with rBASIDIN is an important result since the physiological quality of seeds is influenced by the integrity of the cell membrane system. For example, Chomontowski and Podlaski (2020) reported that the application of solid matrix *priming* and zeolites to sugar beet seeds reduced the electrical conductivity and enhanced the germination capacity of the seeds four days after sowing. In addition, they found that seeds with low vigor had 58% higher electrical conductivity than seeds with high vigor.

In the treatments of lettuce seeds with rBASIDIN, an association between lower electrical conductivity and a higher germination rate at concentrations of 50 and 75 µg mL^-1^ of rBASIDIN occurred. On the other hand, although at a concentration of 100 µg mL^-1^ the seeds suffered lower electrolyte leakage in relation to the controls, they also had lower germination percentage. One hypothesis to explain this finding is that rBASIDIN at this concentration may negatively affect water absorption by seed tissues, hindering or preventing the start of the germination process and compromising radicle emergence (Chomontowski and Podlaski, 2020; Adetunji et al., 2021).

However, the results of the electrical conductivity test indicated that the protein effector rBASIDIN at concentrations of 50 and 75 µg mL^-1^ does not impair the physiological quality of lettuce seeds.

### 4.4 rBASIDIN induces defense-related enzyme activity in lettuce seedlings

The plant defense system can be activated by elicitors, and effectors can generate an elicitor response (Martínez et al., 2024). In plant biology, elicitors are considered extrinsic or foreign molecules that act at very low concentrations and induce defense responses in plants (Vázquez-Hernández et al., 2019). They can bind to specific receptor proteins located in plant cell membranes and are highly specific in the production of secondary metabolites (Lavanya et al., 2022). In some cases, effectors can generate a *priming* effect in plants, whereby a previous treatment can improve the plant’s response to future challenges. This activation process constitutes a type of biological memory, where the plant remembers the initial exposure to stress and uses this to strengthen its defenses (Yang et al., 2022). In view of this, we investigated the protective effect of the effector rBASIDIN when applied to lettuce seeds, evaluating the activity of enzymes related to plant defense.

The chitinases evaluated exhibited different behaviors according to the data shown in **Figures 6A and B**. For exochitinase activity, a statistically significant difference was observed in seedlings treated with rBASIDIN at a concentration of 75µg mL^-1^ in relation to the other treatments, with activity approximately twice as great as the two controls used (**Figure 6A**). However, the endochitinase activity in the seedlings was negatively influenced by the controls when using rBASIDIN at concentrations of 75 and 100 µg mL^-1^, and the other treatments did not differ from each other (**Figure 6B**). According to Kasprzewska (2003), the type of chitinase produced during resistance induction is closely related to the stimulus to which the plant is subjected, which leads us to believe that the type of chitinase evaluated may have varied based on the inducer used.

Chitinases belong to the category of pathogen response proteins, since they directly attack the structural components of insects and fungi (Shoresh and Harman, 2008). The type of action of exokinase is through the hydrolysis of chitin into oligosaccharides. Endochitinases cleave the chitin chain randomly, releasing a mixture of soluble oligomers predominantly diacetylchitobioses (GlcNac)_2_ (Shoresh and Harman, 2008; De Oliveira et al., 2024).

There are reports that chitinase induction in plants is generally nonspecific and increases under biotic and abiotic stresses. In addition, they can accumulate gradually or systematically in other tissues (Vaghela et al., 2022). In this context, we suggest that the higher level of exochitinase in seedlings treated with rBASIDIN, and the statically equal increase in relation to the controls of the endochitinase enzyme in seedlings emerged from seeds treated with rBASIDIN at concentrations of 50 and 75 µg mL^-1^ may increase the protection of the lettuce plants in case they suffers any biotic or abiotic stress. Plants that express both enzymes simultaneously are more resistant than plants that express only one of the enzymes at the same level, since the action of the two enzymes together has a synergistic effect that improves the plant’s defense (Bolar et al., 2001).

The activity of the enzyme β-1-3 glucanase was positively influenced in seedlings from seeds treated with rBASIDIN at a concentration of 75 µg mL^-1^, with the other treatments being the same as the controls (**Figure 6C**). Increased β-1-3 glucanase activity in seedlings may indicate activation of defense mechanisms, since this enzyme is part of the group of proteins related to defense processes during pathogenesis. This enzyme acts by degrading the pathogen’s cell wall, limiting its spread (Perrot et al., 2022). Furthermore, this type of enzyme plays important roles in cell division, trafficking of materials through plasmodesmata and resistance to abiotic stresses, and is also involved in the formation of flowers until seed maturation (Balasubramanian et al., 2012). In this sense, seedlings of *Pennisetum glaucum* 7042S (susceptible cultivar) originating from seeds treated with different elicitors presented significantly higher β-1-3 glucanase activity compared to the controls, when inoculated with the pathogen *Sclerospora graminicola* (Sacc.) (Lavanya et al., 2022).

Although the main interest in β-1-3 glucanases is related to their possible role during plant-pathogen interactions, these enzymes are also involved in several physiological and developmental processes of plants, such as cell division, seed germination, embryogenesis, fruit ripening and the control of callose degradation in plasmodesmata (Perrot et al., 2022; Lyalina et al., 2023).

Another enzyme analyzed by us was catalase, an antioxidant enzyme responsible for removing excess reactive oxygen species (ROS) during stress and for breaking down peroxide into oxygen and water (Billah et al., 2021). In lettuce seedlings, catalase activity had a marked increase when rBASIDIN was applied to the seeds at a concentration of 100 µg mL^-1^. Interestingly, excess ROS can compromise germination and seedling establishment (Soares et al., 2020), so increased activity of catalase may help maintain a strictly regulated concentration of ROS, to improve the adaptive response of lettuce seedlings grown from seeds treated with rBASIDIN. It is worth noting that even with the marked increase in the activity of the antioxidant enzyme catalase at this concentration, this occurrence demonstrated the inefficiency of this enzyme in the detoxification process of the seedling tissues, as evidenced by the reduction in the number of normal seedlings, decreasing the germination rate (**Figure 1**).

Thus, we hypothesize that the ROS accumulation exceeded the scavenging potential of antioxidants and regulation of only a few components of the antioxidant system may not be sufficient to detoxify superoxide radicals. In this sense, it was shown that despite the high activity of superoxide dismutase and peroxidase enzymes, ROS concentrations (O^2-^ and H_2_O_2_) were considerably higher in in Choysum (*Brassica rapa* var. *parachinensis*) seedlings coming from seed treatment with NaCl (Kamran et al., 2021). This was associated with a sharp decline in germination rate, seed germination index and inhibition of seedling growth.

Therefore, because the rBASIDIN effector from seed treatment promotes the activation of the metabolic pathways of enzymes involved in the plant defense mechanism, these results are satisfactory and can be explored in lettuce protection induction programs.

## 5 Conclusion

The present study demonstrated that treatment of seeds with lower concentrations of rBASIDIN had a positive effect on the physiological and biochemical quality of lettuce seeds compared to higher concentrations and controls tested. Therefore, we can conclude that the rBASIDIN effector has a positive effect on germination and development of lettuce seedlings. Furthermore, under the conditions tested, with application of the rBASIDIN effector via lettuce seeds, a positive change was observed in the activity of enzymes related to the plants’ defense response. The results found in this work can be explored in future studies involving biotechnological treatments to induce resistance and promote growth of lettuce plants.

## Data Availability

The original contributions presented in the study are included in the article/supplementary material. Further inquiries can be directed to the corresponding authors.
